# Comprehension of an artificial intelligence-generated discharge letter versus a traditional discharge letter: two controlled quasi-experimental parallel-group studies

**DOI:** 10.1093/intqhc/mzag133

**Published:** 2026-09-12

**Authors:** Fabrizio Bert, Lorenzo Rosset, Francesco Conrado, Daniele Consoli, Giacomo Scaioli, Giuseppina Lo Moro, Roberta Siliquini

**Affiliations:** Department of Public Health and Pediatric Sciences, University of Turin, Turin, Italy; Department of Public Health and Pediatric Sciences, University of Turin, Turin, Italy; Department of Public Health and Pediatric Sciences, University of Turin, Turin, Italy; Department of Public Health and Pediatric Sciences, University of Turin, Turin, Italy; Department of Public Health and Pediatric Sciences, University of Turin, Turin, Italy; Department of Public Health and Pediatric Sciences, University of Turin, Turin, Italy; Department of Public Health and Pediatric Sciences, University of Turin, Turin, Italy; AOU City of Health and Science of Turin, Turin, Italy

**Keywords:** discharge letter, artificial intelligence, health literacy, patient comprehension, hospital discharge, patient communication, patient discharge summaries, generative artificial intelligence

## Abstract

**Background:**

Discharge letters are essential for continuity of care, yet patients often misunderstand their content. Poor discharge communication is associated with medication errors, inappropriate healthcare use, and hospital readmissions. Artificial intelligence (AI) language models may improve discharge documentation clarity and accessibility. This study aimed to compare a GPT-4-generated, accessibility-optimized discharge letter with a conventional discharge letter regarding comprehension of key discharge information and to evaluate satisfaction and identify predictors of comprehension.

**Methods:**

Two controlled quasi-experimental parallel-group studies were conducted. Study 1 included adults recruited online (*n* = 341 analysed), while Study 2 involved medical and nursing students at the University of Turin (*n* = 791 analysed). Allocation was based on age parity. The primary outcome was a structured comprehension score assessing diagnosis, treatment, investigations, and follow-up instructions. Secondary outcomes included readability, clarity, perceived comprehension, organization, and satisfaction. Health literacy (HL) was assessed using the HLS-EU-Q6. Multiple linear regression analyses identified predictors of comprehension.

**Results:**

Participants receiving the AI-generated discharge letter achieved significantly higher comprehension scores in both studies (Study 1: median 4 vs. 2; Study 2: median 4 vs. 3; both *P* < .001). Differences remained significant after adjustment for sociodemographic variables, HL, and healthcare background. AI-generated letters also improved all secondary outcomes (all *P* < .001). No significant improvement was observed for identification of follow-up appointment dates. Higher comprehension was associated with healthcare background, higher education, sufficient HL, and better perceived health status.

**Conclusion:**

AI-generated discharge letters significantly improved measured comprehension and satisfaction in both general and higher-literacy populations; validation using real clinical letters and diverse patients is required.

## Introduction

The transition from hospital to community care is a high-risk phase in which communication failures are common and clinically important [1–3]. Although discharge letters support continuity of care by summarizing hospital stays, treatments, investigations, and follow-up recommendations [1, 2], they often communicate poorly with patients.

Patients frequently misunderstand diagnoses, medications, follow-up plans, and warning signs [4, 5]. Poor understanding has been linked to medication errors, unplanned healthcare use, emergency department visits, and readmissions [6–8], particularly among people with limited health literacy (HL) or proficiency in the document language [9].

Discharge letters must be clinically complete, clear, and accessible [2, 10]. Core features include diagnoses, treatments, reasons for medication changes, key test results, and follow-up instructions [11], but these elements can increase linguistic and structural complexity.

Some approaches include plain-language rewriting [10, 12] and teach-back [13] but may be resource-intensive, time-consuming, and difficult to standardize at scale [8, 12].

Advances in artificial intelligence (AI) may offer a complementary solution. Large language models (LLMs) may simplify text, reorganize information, and adapt content to different literacy levels [14]. Their potential for patient engagement, health communication, and accessibility is increasingly being studied [15, 16]. AI-generated letters may therefore offer a scalable way to improve clarity without reducing clinical completeness.

Therefore, our primary objective was to compare an AI-generated discharge letter optimized for accessibility with a traditional discharge letter in terms of comprehension of key discharge information. To address variability in HL and medical knowledge, the intervention was examined in two complementary populations: a general adult population (Study 1) and a higher-literacy population consisting of healthcare students (Study 2). Secondary objectives were to assess satisfaction with the discharge letters and to identify variables associated with comprehension. We hypothesized that participants receiving the AI-rewritten letter would achieve higher comprehension scores and report greater perceived satisfaction than those receiving the original synthetic letter in both studies.

## Materials and methods

### Study design

This research consisted of two controlled quasi-experimental parallel-group studies, conducted in accordance with the TREND statement for nonrandomized evaluations [17]. Ethical approval was obtained from the Ethics Committee of the University of Turin (protocol 0290583, 6 July 2023). Participation was voluntary, anonymous, and uncompensated.

### Participants

Study 1: Participants were adults recruited through a digital dissemination strategy using social media. The questionnaire was administered via LimeSurvey. Recruitment and data collection took place between March and July 2023.

Study 2: Participants were medical and nursing students at the University of Turin, recruited during classes. Recruitment and data collection occurred between September and November 2023.

In both studies, inclusion criteria required participants to be at least 18 years of age and proficient in Italian. Exclusion criteria included missing age information and failure to complete the questionnaire section assessing comprehension of the letter.

### Intervention

A synthetic discharge letter written in Italian was developed to ensure patient privacy and to avoid ethical concerns associated with real clinical documentation use. The document was designed to closely resemble a standard hospital discharge letter, including a summary of the hospitalization, diagnostic investigations performed, treatments administered, medications prescribed at discharge, and post-discharge recommendations.

To generate an accessibility-optimized version of the letter, a structured prompt was developed to instruct GPT-4 (legacy model), an LLM developed by OpenAI [14], to rewrite the original discharge letter using simpler language, clearer organization, and explicit explanations, while preserving clinically relevant content. The AI-generated output was used without manual modification to assess autonomous model performance.

Participants in the intervention group received the AI-generated discharge letter, whereas participants in the control group received the original discharge letter. The intervention differed slightly between the two studies to reflect differences in participant characteristics and study aims. Details about the letter development are reported in Supplementary Methods M1.

A content-fidelity assessment by two authors who were medical doctors showed 81.8% agreement for accuracy (κ = 0.00 owing to imbalanced marginals), 90.9% for completeness (κ = 0.62), and 100% for the absence of hallucinated, fabricated, or unsupported additional content (κ not estimable) (Supplementary Methods M2). AI-generated letters were shorter than the original versions (365 vs. 388 words in Study 1; 814 vs. 1024 in Study 2), with comparable Gulpease indices [18] (48.6 vs. 49.9 and 49.1 vs. 48.7, respectively; Supplementary Methods M3).

### Measurements and outcomes

The discharge letter was accompanied by a questionnaire of four sections.

The first section collected sociodemographic information, including age, gender, nationality, and mother tongue; the latter included to assess the potential impact of language proficiency on comprehension. Educational level and perceived socioeconomic status were collected as established HL determinants [19, 20]. Participants also reported healthcare-related experience (current studies, employment, or past professional experience) as a proxy for familiarity with medical terminology. Self-rated health status and healthcare service utilization were included to explore their association with discharge letter comprehension. In Study 2, participants additionally reported their degree programme and year of study.

The second section assessed HL using the six-item European Health Literacy Survey Questionnaire (HLS-EU-Q6) [21]. The HLS-EU-Q6 has been validated in the Italian context [22]. The overall HLS-EU-Q6 score was calculated as the mean of item scores. HL levels were categorized according to established thresholds: inadequate (≤2), problematic (>2 and ≤3), and sufficient (>3).

The third section assessed the primary outcome, i.e. comprehension of the discharge letter, designed with reference to the methodology proposed by Lin *et al.* [23] and aimed to evaluate participants’ understanding of key information commonly included in high-quality hospital discharge letters [23], including diagnosis, treatments, diagnostic investigations, and follow-up planning [2, 10]. The comprehension score ranged from 0 to 5 in Study 1 and from 0 to 6 in Study 2 (Supplementary Methods M4).

The final section assessed perceived comprehension and satisfaction with the discharge letter, modelled on the approach described by Smolle *et al.* [10]. Participants rated their agreement with seven statements (Supplementary Methods M5). Responses were recorded using a five-point Likert scale ranging from 1 (strongly disagree) to 5 (strongly agree). For both Study 1 and 2, these seven items represented the secondary outcomes.

### Sample size

The sample size was defined to detect a minimum clinically meaningful difference of 10% in comprehension between study arms, with a two-sided confidence level of 95% and a statistical power of 80%. Based on prior evidence by Lin *et al.* [23], a baseline comprehension score of 3 with a SD of 1.5 on a 0–5 scale was assumed. A target sample size of approximately 150 participants per group was established for each study.

### Assigned method

After providing informed consent and accessing the questionnaire, participants were automatically allocated to either the intervention or control group according to a predefined deterministic rule based on age parity. Group assignment was determined by the parity of the participant’s age (odd vs. even), a predefined and automated rule chosen to ensure an approximately balanced distribution across study arms while maintaining feasibility within an online survey framework.

### Blinding

Participants were blinded to group allocation and were not informed of the existence of two different versions of the discharge letter. Participants were unaware of the involvement of AI in the generation of the material. Blinding of the investigators was not applicable.

### Unit of analysis and statistical methods

The individual participant was the unit of both assignment and analysis.

Descriptive analyses were performed. Continuous variables were summarized using medians and interquartile ranges (IQRs) due to non-normal distributions (Shapiro–Wilk test). Between-group comparisons of baseline characteristics were performed using chi-square tests and Mann–Whitney *U* tests.

Given 25.9% missing comprehension data in Study 1, missingness rates were compared between groups using a chi-square test and unadjusted extreme-case bounds were calculated by assigning missing scores of 0 or 5 in opposite directions.

The primary outcome (comprehension score) was compared between intervention and control groups using the Mann–Whitney *U* test. For each individual item of the comprehension score, chi-square tests were conducted to compare the proportion of correct responses between groups. To assess the association between letter type and comprehension after accounting for participant characteristics, multiple linear regression models were fitted. Covariates included sociodemographic variables, HL level, and healthcare-related background. Residual normality was confirmed visually (Supplementary Methods M6), and robustness was evaluated using bias-corrected 95% CIs from 5000 nonparametric bootstrap replications.

Separate multiple linear regression models were conducted within the intervention and control groups to explore differences in predictors of comprehension.

Secondary outcomes were compared between study arms using Mann–Whitney *U* tests. *P*-value for the seven secondary outcomes were adjusted separately within each study using the Holm–Bonferroni procedure.

Additional analyses were performed. In Study 1, comprehension scores were compared between participants with and without a healthcare background within each study arm using Mann–Whitney *U* tests. Also, multivariable linear regression models stratified by healthcare background were fitted, adjusting for the same covariates as the main model.

In Study 2, separate multiple linear regression models were fitted for medical students and nursing students to assess whether different variables influenced comprehension.

Statistical analyses were conducted using RStudio (version 2023.09.0 + 463) and Stata (version 18 and 19). A two-sided significance level of .05 was adopted. Missing data were handled using complete-case analysis.

## Results

### Study 1: General population

#### Flow and baseline characteristics

A total of 225 participants were assigned to the control group and 235 participants to the intervention group. Following exclusions for an incomplete third survey section, 166 participants from the control group and 175 from the intervention group were included (flow in Supplementary Fig. S1). Missingness was similar in the traditional- and AI-letter groups (26.2% vs. 25.5%; *P* = .866).

Baseline characteristics of the two arms are reported in Supplementary Table SI. No significant differences were observed, except working (or studying) as a healthcare professional, which was more frequent in the intervention group (*P* = .041).

#### Primary outcome

The median comprehension score of the control group was 2 (IQR = 1–2), significantly lower (*P* < .001) than that of the intervention group, which was 4 (IQR = 2–4). The complete-case unadjusted mean difference was 1.52 points; extreme-case bounds ranged from −0.17 to 2.42, with the lower bound requiring all missing AI scores to be 0 and all missing traditional-letter scores to be 5.

A detailed description of each item of the primary outcome stratified by the two arms is presented in Supplementary Table SII. The intervention group showed higher comprehension of all items except for the identification of the follow-up appointment date (*P* = .160).

The multiple regression (Table 1) performed on the whole sample indicated that the intervention group had significantly higher comprehension scores after adjustment. The bootstrap analysis confirmed this association [adjusted coefficient = 1.379, bias-corrected 95% confidence interval (CI) 1.144–1.608]. Other significant predictors included gender, education, healthcare background, and sufficient HL.

**Table 1:** Multiple linear regression model for the primary outcome (comprehension score) (Study 1).

	Adjusted estimate	Standard error	Lower 95% CI	Upper 95% CI	*P*-value
Group					
Control group	Ref.				
Intervention group	1.384	0.229	1.156	1.613	**<.001**
Age (years)	0.003	0.008	−0.005	0.011	.409
Gender					
Female	Ref.				
Male	0.388	0.252	0.135	0.640	**.003**
Mother tongue					
Italian	Ref.				
Other	−0.177	0.550	−0.727	0.373	.527
Education level					
Middle school or below	Ref.				
High school diploma	0.268	0.512	−0.244	0.780	.304
Bachelor’s degree	0.596	0.553	0.043	1.149	**.035**
Master’s degree or above	0.625	0.551	0.073	1.176	**.027**
Working, studying or have worked as a healthcare professional					
No	Ref.				
Yes	0.815	0.273	0.541	1.088	**<.001**
Perceived economic situation					
Poor or insufficient	Ref.				
Excellent or good	−0.133	0.368	−0.501	0.235	.476
Perceived health status					
Poor or insufficient	Ref.				
Excellent or good	0.371	0.347	0.024	0.718	**.036**
Healthcare services usage					
Nonusers (<1 times per year)	Ref.				
Users (at least 1–2 times per year)	0.053	0.257	−0.204	0.310	.686
Health literacy score					
Inadequate	Ref.				
Problematic	0.259	0.298	−0.039	0.557	.088
Sufficient	0.371	0.347	0.024	0.718	**<.001**

Note: Bold values indicate statistically significant P-values (P < 0.05).

Considering the model including only the intervention group (Supplementary Table SIII), the analysis revealed several predictors. Males demonstrated higher comprehension. Individuals holding a bachelor’s or a master’s degree showed higher comprehension than those with a middle school education. A healthcare background, sufficient HL, and perceived better health status predicted higher comprehension.

In the control group model (Supplementary Table SIV), the significant predictors were different. Gender, education level, and perceived economic situation had no significant association. Similar to the intervention group model, healthcare background and sufficient HL were strong positive predictors. Non-Italian speakers showed significantly lower comprehension.

In the subgroup analysis, significant differences were observed when comparing healthcare professionals with non-healthcare individuals (Fig. 1). For the AI-generated letter, people with a healthcare background had a median comprehension score of 4 (IQR = 3–4) and non-healthcare individuals a median score of 3.5 (IQR = 2–4) (*P* = .263). In the original letter group, healthcare professionals demonstrated a significantly higher comprehension, with a median score of 3 (IQR = 2–3), compared to non-healthcare individuals, who had a median score of 1 (IQR = 1–2) (*P* < .001). A cross-group comparison showed a non-significant trend towards higher comprehension (*P* = .052). In the multivariable models stratified by healthcare background (Supplementary Tables SV and SVI), assignment to the AI-generated letter remained significantly associated with higher comprehension both among non-healthcare individuals (adjCoef = 1.667) and among healthcare professionals (adjCoef = 0.458).

**Figure 1:**
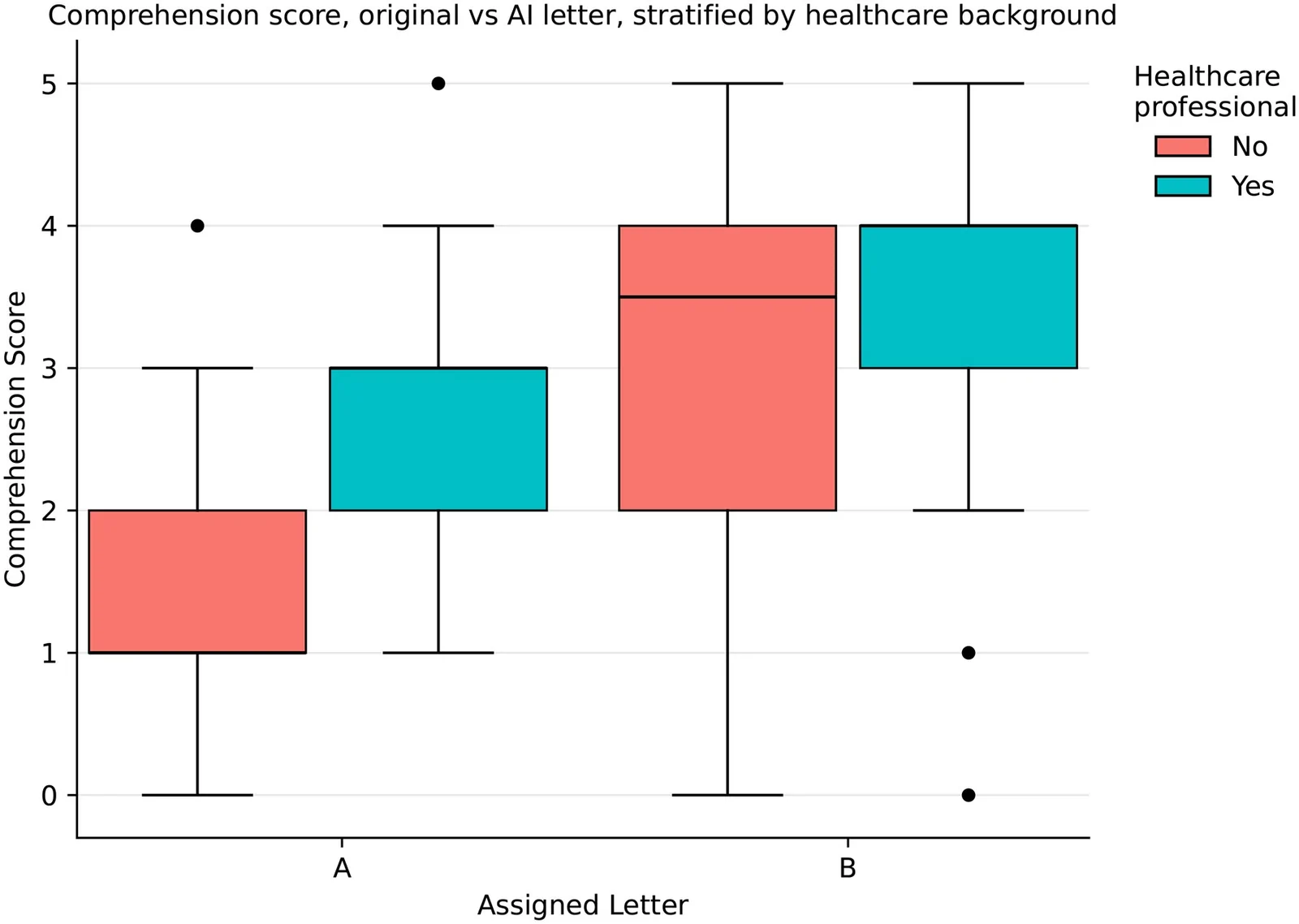
Boxplot of comprehension scores stratified by healthcare background and study group (Study 1). Panel A shows scores for participants who received the original discharge letter; while Panel B shows scores for participants who received the AI-generated discharge letter. In each panel, the left box represents participants without a healthcare background and the right box represents participants with a healthcare background. The horizontal line within each box represents the median; the box spans the IQR; whiskers extend to ×1.5 the IQR; individual points represent outliers.

#### Secondary outcomes

Secondary outcomes are presented in Table 2. The intervention group reported higher satisfaction and perceived comprehension regarding all secondary outcomes (*P* < .001).

**Table 2:** Secondary outcomes stratified by experimental and control groups (Study 1).

Secondary outcomes	Arms of the study	Median (IQR)	Mann–Whitney *U* test *P*-value^a^
Ease of reading and understanding the discharge letter	Original letter	1 (1–2)	<.001
	AI-generated letter	4 (3–5)	
Organization of information in the discharge letter	Original letter	2 (2–3)	<.001
	AI-generated letter	4 (4–5)	
Clarity in addressing doubts and concerns	Original letter	2 (1–2)	<.001
	AI-generated letter	4 (2–4)	
Presence of complex and unclear words	Original letter	5 (4–5)	
	AI-generated letter	2 (2–4)	<.001
Ability to follow post-discharge instructions without seeking further explanation	Original letter	2 (1–3)	<.001
	AI-generated letter	4 (3–5)	
Overall satisfaction with the discharge letter	Original letter	2 (1–3)	<.001
	AI-generated letter	4 (3–4)	
Preference for a more easily understandable version of the letter (higher scores indicate a stronger preference for an alternative, more easily understandable version of the discharge letter)	Original letter	5 (4–5)	<.001
	AI-generated letter	3 (2–4)	

a *P*-value with Holm–Bonferroni correction.

### Study 2: Students in health-related fields

#### Flow and baseline characteristics

A total of 455 participants were assigned to the control group, while 429 were assigned to the intervention group. Only participants who completed the third questionnaire section were included: 407 participants from the control group and 384 participants from the intervention group (flow in Supplementary Fig. S2).

Supplementary Table SVII presents the baseline characteristics. No significant differences were observed between groups.

#### Primary outcome

The experimental group reported a median comprehension score of 4 (IQR = 3–5) and the control group a median score of 3 (IQR = 2–4). (*P* < .001). When examining individual comprehension items (Supplementary Table SVIII), significant differences were observed except for identifying scheduled follow-up appointments (*P* = .422).

The multiple regression performed on the whole sample (Table 3) highlighted that the intervention group showed significantly higher scores than the control group. The bootstrap analysis confirmed this association (adjusted coefficient = 0.919, bias-corrected 95% CI 0.744-1.082). Lower scores were observed in males and nursing students. Third-year students had higher scores than first-year students, and frequent healthcare users scored slightly higher.

**Table 3:** Multiple linear regression model for the primary outcome (comprehension score) (Study 2).

	Adjusted estimate	Standard error	Lower 95% CI	Upper 95% CI	*P*-value
Group					
Control group	Ref.				
Intervention group	0.920	0.087	0.750	1.090	**<.001**
Age (years)	−0.002	0.013	−0.028	0.025	.896
Gender					
Female	Ref.				
Male	−0.253	0.102	−0.453	−0.052	**.014**
Nationality					
Italian	Ref.				
Other	−0.245	0.350	−0.932	0.457	.484
Mother tongue					
Italian	Ref.				
Other	−0.000	0.340	−0.667	0.667	.999
Degree course					
Medicine and surgery	Ref.				
Nursing	−0.512	0.091	−0.691	−0.33	**<.001**
Year of study					
1	Ref.				
2	0.095	0.100	−0.100	0.291	.339
>3	0.698	0.124	0.455	0.941	**<.001**
Perceived economic situation					
Poor or insufficient	Ref.				
Excellent or good	−0.156	0.143	−0.437	0.124	.275
Perceived health status					
Poor or insufficient	Ref.				
Excellent or good	0.160	0.176	−0.186	0.506	.364
Healthcare services usage					
Nonusers (<1 times per year)	Ref.				
Users (at least 1–2 times per year)	0.205	0.094	0.020	0.389	**.030**
Health literacy score					
Inadequate	Ref.				
Problematic	−0.067	0.172	−0.404	0.270	.695
Sufficient	−0.007	0.213	−0.425	0.412	.975

Note: Bold values indicate statistically significant P-values (P < 0.05).

For the intervention group (Supplementary Table SIX), the multiple linear regression revealed several significant predictors. Nursing students had lower comprehension. Third-year students showed better comprehension than first-year students. A perceived better health status also predicted higher comprehension. Users of healthcare services demonstrated better comprehension.

In the control group (Supplementary Table SX), the results showed fewer significant predictors. Similar to the intervention group, third-year students outperformed first-year students, and nursing students had lower comprehension scores.

In the multiple regression conducted on the subgroup of medical students (Supplementary Table SXI), the intervention group exhibited significantly higher scores. Male students showed slightly lower scores, while third-year students had higher scores than first-year students.

Consistent with the findings in medical students, the multiple regression for nursing students (Supplementary Table SXII) showed that the intervention group achieved significantly higher comprehension scores. Higher scores were observed in second-year and third-year students compared to first-year students.

#### Secondary outcomes

Secondary outcomes (Table 4) showed that the AI-generated discharge letter outperformed the original version across all aspects (*P* < .001).

**Table 4:** Secondary outcomes stratified by experimental and control groups (Study 2).

Secondary outcomes	Arms of the study	Median (IQR)	Mann–Whitney *U* test *P*-value^a^
Ease of reading and understanding the discharge letter	Original version	2 (2–3)	<.001
	AI-generated version	4 (3–4)	
Organization of information in the discharge letter	Original version	3 (2–4)	<.001
	AI-generated version	3 (3–4)	
Clarity in addressing doubts and concerns	Original version	3 (2–3)	<.001
	AI-generated version	3 (3–4)	
Presence of complex and unclear words	Original version	4 (3–5)	<.001
	AI-generated version	3 (2–4)	
Ability to follow postdischarge instructions without seeking further explanation	Original version	2 (2–3)	<.001
	AI-generated version	3.5 (3–4)	
Overall satisfaction with the discharge letter	Original version	3 (2–3)	<.001
	AI-generated version	4 (3–4)	
Preference for a more easily understandable version of the letter (higher scores indicate a stronger preference for an alternative, more easily understandable version of the discharge letter)	Original version	4 (3–5)	<.001
	AI-generated version	3 (2–4)	

a *P*-value with Holm–Bonferroni correction.

## Discussion

### Statement of principal findings

This study compared comprehension and perceived satisfaction between an AI-optimized and a traditional discharge letter in the general population (Study 1) and healthcare students (Study 2). In both studies, the AI-generated letter improved measured comprehension and satisfaction, providing preliminary evidence that the use of AI language models may make discharge communication more accessible.

The only domain that did not differ between study arms across both studies was the identification of the follow-up appointment date. This finding likely reflects that follow-up information was clearly and explicitly stated in both versions of the letter, indicating a ceiling effect for this specific item.

### Interpretation within the context of the wider literature

The observed improvements align with prior literature showing that patients frequently misunderstand discharge instructions and may be unaware of these comprehension gaps [5]. Previous studies have demonstrated that improving the readability and clarity of discharge documentation is associated with better understanding [23], reduced post-discharge queries, and lower rates of unplanned healthcare utilization [24]. Our findings extend this evidence by supporting further evaluation of AI-assisted rewriting in real clinical settings.

Beyond comprehension, participants receiving the AI-generated discharge letter reported higher satisfaction and perceived understanding across all dimensions in both studies. They found it easier to read, better organized, clearer in addressing doubts, and easier to follow without extra explanation, with less complex terminology. Control group participants showed a stronger preference for a simpler discharge letter, suggesting that clearer communication may reduce unmet informational needs and perceived gaps in understanding.

Regression analyses provided additional insights into factors associated with discharge letter comprehension. Higher education, healthcare background, sufficient HL, and better perceived health status were consistently associated with higher comprehension scores.

These findings are coherent with existing conceptual models of HL, which posit that the ability to access, understand, appraise, and apply health information directly influences engagement with healthcare and health outcomes [19, 25, 26].

In Study 1, lower comprehension among non-Italian speakers was observed in the control group but was weaker in the intervention group, suggesting that the AI-generated letter may partly reduce language-related barriers through simpler syntax and clearer structure. A smaller comprehension gap between participants with and without a healthcare background was observed in the AI-letter group: lay participants who read the AI-generated letter achieved scores comparable to healthcare professionals who read the original letter, suggesting that AI-based simplification may broaden access to medical information by reducing reliance on prior clinical knowledge.

In Study 2, comprehension varied according to academic background and progression. Medical students outperformed nursing students, and students in more advanced years showed higher comprehension than those in earlier years. These differences may reflect variations in curricular emphasis and exposure to medical documentation. In the Italian academic system, medical students typically enter structured clinical training in their third year, whereas nursing students are exposed to clinical environments earlier but may engage less extensively with physician-authored discharge documentation. Despite these differences, higher comprehension scores were observed in the AI-letter group across the subgroups, including both medical and nursing students. This suggests that simplification and reorganization strategies may also benefit individuals with formal healthcare training. HL differed substantially between the general population and student cohorts, with lower levels observed in the former. This is consistent with previous evidence indicating that average HL levels in Italy are often below those required for full engagement with complex health materials [27–29]. The intervention improved comprehension across both cohorts, underscoring its adaptability across a wide spectrum of HL levels and reinforcing the potential role of AI-enhanced communication as a complementary strategy to address HL-related disparities.

### Strengths and limitations

This study has several strengths. It examined two complementary populations, increasing the breadth of evidence. Participant blinding, automated allocation, and the use of a controlled design reduce key sources of bias. The comprehension instrument covered multiple clinically relevant domains.

Several limitations should be considered. Using a synthetic letter describing a case of chronic obstructive pulmonary disease (COPD) in Italian limits generalizability to real-world documents, other conditions, languages, and healthcare systems. Online recruitment and the student sample may have introduced selection bias and under-represented vulnerable populations. Deter­ministic, unconcealed age-parity allocation provides less internal validity than randomization; despite adjusted and stratified analyses, residual confounding remains possible. Attrition bias cannot be excluded because individual-level data were unavailable and extreme-case bounds included the null. The comprehension instruments were not pilot-tested or psychometrically validated, and open-ended responses were scored without blinding or quantified inter-rater agreement. Exact regeneration was precluded by the proprietary GPT-4 interface and unavailable model parameters. Reproducibility was further constrained by GPT-4-0314’s subsequent retirement and by the inherent non-determinism of LLM outputs, which persists even with identical parameters, meaning exact regeneration would not be guaranteed regardless of documentation completeness. Dedicated reporting guidelines for LLM-based studies, namely TRIPOD-LLM [30] and CHART [31], were published only in 2024–5, after this study was designed and conducted, and could therefore not be applied prospectively; their principles should inform future reporting of model version, prompts, and generation parameters in this field. The fidelity assessment was limited to comprehension domains and conducted by two nonspecialist physician authors. Comparable Gulpease scores despite shorter AI letters also suggest that this index may not capture organizational improvements. Finally, no validated minimal clinically important difference was available, and medication errors, self-management, and readmissions were not assessed; clinical significance remains uncertain and real-world validation is required before implementation.

### Implications for policy, practice, and research

Our findings support further evaluation of AI-assisted discharge letter rewriting rather than immediate clinical implementation. Regulatory frameworks addressing accountability and reproducibility of LLMs in clinical communication should be developed. Future research should assess real-world outcomes including medication adherence, readmissions, and healthcare utilization across multiple languages and clinical conditions.

## Conclusions

AI-generated, accessibility-optimized discharge letters may ­significantly improve comprehension and satisfaction across both lay and higher-literacy populations. Future research should ­evaluate these interventions in real clinical settings and assess downstream outcomes including adherence, healthcare utilization, and patient safety.

## Supplementary Material

mzag133_Supplementary_Data

## Data Availability

The data underlying this article are not publicly available due to privacy considerations but may be made available upon reasonable request to the corresponding author (D.C., daniele.consoli@unito.it).
