# Supplementary material for: Comprehension of an artificial intelligence-generated discharge letter versus a traditional discharge letter: two controlled quasi-experimental parallel-group studies
Source: Int J Qual Health Care. 2026 Sep 12;38(4):mzag133. doi: 10.1093/intqhc/mzag133 (PMC13659911; doi:10.1093/intqhc/mzag133)
Supplement: Supplementary file 1 [file mzag133_supplementary_data.docx]

**SUPPLEMENTARY FILE**

**Comprehension of an AI-generated discharge letter versus a traditional discharge letter: two controlled quasi-experimental parallel-group studies**

Fabrizio Bert, MD Prof¹, Lorenzo Rosset, MD¹, Francesco Conrado, MD¹, Daniele Consoli, MD¹*, Giacomo Scaioli, MD¹, Giuseppina Lo Moro, MD¹, Roberta Siliquini, MD Prof¹^,^²

*Corresponding author: Daniele Consoli, MD, daniele.consoli@unito.it, Via Santena 5/bis, Torino, Italy

Affiliations:

1.	Department of Public Health, University of Turin, Turin, Italy

2.	AOU City of Health and Science of Turin, Turin, Italy

Table of contents

[SUPPLEMENTARY METHODS	3](#_Toc237014519)

[**M1.** Development of the synthetic discharge letter and ai rewriting	3](#_Toc237014520)

[**M2.** Clinical content-fidelity assessment	10](#_Toc237014521)

[**M3.** Quantitative readability and document length	10](#_Toc237014522)

[**M4.** Comprehension assessment	11](#_Toc237014523)

[**M5.** Secondary outcome measures	11](#_Toc237014524)

[**M6.** Residual normality check	11](#_Toc237014525)

[SUPPLEMENTARY FIGURES	13](#_Toc237014526)

[**Fig. S1.** Flow diagram of the recruitment process (Study 1)	13](#_Toc237014527)

[**Fig. S2.** Flow diagram of the recruitment process (Study 2)	14](#_Toc237014528)

[14](#_Toc237014529)

[SUPPLEMENTARY TABLES	15](#_Toc237014530)

[**Table SI** Baseline socio-demographic characteristics and health literacy scores stratified by experimental and control groups (Study 1)	15](#_Toc237014531)

[**Table SII** Differences in items composing the primary outcome between intervention and control groups (Study 1)	16](#_Toc237014532)

[**Table SIII** Multiple Linear Regression Model of Intervention group – AI-generated version of the discharge letter. Dependent variable: comprehension score (Study 1)	17](#_Toc237014533)

[**Table SIV** Multiple Linear Regression Model of Control group – Original version of the discharge letter. Dependent variable: comprehension score (Study 1)	18](#_Toc237014534)

[**Table SV** Multiple linear regression model for the primary outcome (comprehension score), Subsample of non-healthcare professionals. (Study 1)	19](#_Toc237014535)

[**Table SVI** Multiple linear regression model for the primary outcome (comprehension score), Subsample of healthcare professionals. (Study 1)	20](#_Toc237014536)

[**Table SVII** Baseline socio-demographic characteristics and health literacy scores stratified by experimental and control groups (Study 2)	21](#_Toc237014537)

[**Table SVIII** Differences in items composing the primary outcome between intervention and control groups (Study 2)	22](#_Toc237014538)

[**Table SIX** Multiple Linear Regression Model of Intervention group – AI-generated version of the discharge letter. Dependent variable: comprehension score (Study 2)	23](#_Toc237014539)

[**Table SX** Multiple Linear Regression Model of Control group – Original version of the discharge letter. Dependent variable: comprehension score (Study 2)	24](#_Toc237014540)

[**Table SXI** Multiple Linear Regression Model of medical students’ subgroup. Dependent variable: comprehension score (Study 2)	25](#_Toc237014541)

[**Table SXII** Multiple Linear Regression Model of nursing students’ subgroup. Dependent variable: comprehension score (Study 2)	26](#_Toc237014542)

# SUPPLEMENTARY METHODS

## **M1.** Development of the synthetic discharge letter and ai rewriting

**Selected clinical condition:** Chronic Obstructive Pulmonary Disease (COPD) was selected as the reference clinical condition because it represents a common chronic illness frequently associated with multimorbidity, complex pharmacological regimens, and articulated discharge instructions,^1^ making it a suitable model for testing comprehension of discharge documentation.

**AI-Rewritten letter generation:** The AI-rewritten letter was generated on May 2023 using GPT-4 through the ChatGPT web interface with an OpenAI ChatGPT Plus subscription. The API was not used; therefore, no API endpoint was applicable, and the exact backend model snapshot, system prompt, temperature, and maximum token limit were not exposed to the user and cannot be retrospectively verified. At that date the model served by the interface corresponded to the GPT-4 snapshot then in production (gpt-4-0314; the subsequent gpt-4-0613 snapshot was released in June 2023). A single generation was performed, and the output was used without manual modification. Because the gpt-4-0314 snapshot has since been retired, and because large language models are not deterministic, exact regeneration of the output is not possible. The following archived materials (prompts and letters) constitute the record of the intervention.

The intervention differed slightly between the two studies to reflect differences in participant characteristics and study aims. In Study 1, the original letter was shorter to emphasize key information and to reduce respondent burden and dropout in the general population. In Study 2, the full-length version of original discharge letter was used to assess comprehension of more detailed and structurally complex discharge documentation among healthcare students, conceptualized as a higher-literacy population. Consequently, also the AI-rewritten letters differed in length.

**Prompt:**

***Italian version:*** “Riscrivi la seguente lettera di dimissione medica in una versione di facile comprensione per chi non ha una formazione medica. Semplificare la terminologia medica e spiegare qualsiasi procedura o concetto complesso in termini laici. Assicurarsi che tutte le informazioni sanitarie essenziali, le istruzioni e le raccomandazioni siano trasmesse in modo accurato e chiaro, senza omettere alcun dettaglio essenziale per la cura del paziente dopo la dimissione.”

***English version****:* “Rewrite the following medical discharge letter in a version that is easy to understand for those without medical training. Simplify medical terminology and explain any complex procedure or concept in layman terms. Ensure that all essential health information, instructions and recommendations are conveyed accurately and clearly, without omitting any details essential to the patient's care after discharge.”

**Letters:**

**STUDY 1 – GENERAL POPULATION**

**CONTROL LETTER – Study 1**

In PS avviata terapia con amoxicillina/clavulanato, azitromicina, metilprednisolone 80 mg, pantoprazolo, insulina, salbutamolo/ipretropio bromuro, enoxaparina a dosaggio di profilassi e NIV, FR 26, FiO2 41, EPAP 5, IPAP 15 con successiva titolazione.

Ricovero in Pneumologia Semintensiva/degenza ordinaria dal 21/04 al 02/05

All'ingresso in reparto paziente VCO, tachipnoica all'eloquio in VM 50%. All'ECO: sliding presente bilateralmente. Pattern A diffuso. FE iespettiva conservata, camere dx non dilatate, VCI 1.39 collabente > 50%

CUS: asse femoro popliteo comprimibile bilateralmente

Eseguito EGA in MV 50%; ipossiemia moderata, ipercapnia lieve. Si impostavano HFNC 60/0.6.

Durante la degenza si è assistito a un progressivo miglioramento degli scambi respiratori con svezzamento dall'ossigeno terapia ad alti flussi e mantenimento di O2 a bassi flussi. Al momento della dimissione paziente eupnoica in CN 1 1/min (già in possesso di O2 domiciliare).

E' stata proseguita la terapia antibiotica con amoxi/ac. clavulanico dal 21/04 al 28/04. In seguito al riscontro di positività su escreato a Haemophilus influenzae (Carica microbica: 1.000.000 UFC./ml) è stata sostituita con Bactrim per OS (dal 28/04).

La paziente è stata presa in carico dal punto di vista fisioterapico, con ripresa della mobilizzazione in autonomia.

Si dimette la paziente in data odierna, in buone condizioni generali. Parametri vitali stabili

Diagnosi alla dimissione IR acuta su cronica in BPCO riacutizzata con sovrapposizione flogistica (es. batteriologico positivo su espettorato per H. Influenzae)

Prossimi appuntamenti: -Visita pneumologica di controllo in data 5/7/2023 h 11.45

Terapia consigliata:

| Farmaco prescritto | Posologia | Annotazioni medico | Stato prescrizione | Fascia/nota AIFA |
| --- | --- | --- | --- | --- |
| Mattino | | | | |
| Beclometasone + formoterolo 100/6 mcg per erogaz. Soluz. Pressurizzata | 2 DOS |  | Continuazione terapia domiciliare | A |
| Pantoprazolo sale sodico 20 mg 28 cpr gastroresistenti | 1 CPR |  | Nuova prescrizione iniziata in ricovero | A nota 48 |
| Tiotropio bromuro 2,5 mcg soluz per inalazione respirat + 1 cartuccia da 60 erogazioni | 2 DOS |  | Nuova prescrizione iniziata al domicilio | A |
| Simavastatina | 1 CPR |  | Nuova prescrizione iniziata al domicilio | A Nota 13 |
| Colazione | | | | |
| Sulfametoxazolo + trimetoprim 160 mg + 800 mg 16 compresse | 1 CPR | Assumere per 3 gg | Nuova prescrizione iniziata in ricovero | A |
| Cena | | | | |
| Sulfametoxazolo + trimetoprim 160 mg + 800 mg 16 compresse | 1 CPR | Assumere per 3 gg | Nuova prescrizione iniziata in ricovero | A |
| Metformina cloridrato 500 mg 30 cpr rivestite | 1 CPR |  | Nuova prescrizione iniziata al domicilio | A |
| Sera | | | | |
| Beclometasone + formoterolo 100/6 mcg per erogaz. Soluz. Pressurizzata | 2 DOS |  | Continuazione terapia domiciliare | A |

**ARTIFICIAL INTELLIGENCE LETTER – Study 1**

In Pronto Soccorso, la sig.ra ROSSI ha iniziato un trattamento con:

Amoxicillina/clavulanato e azitromicina (antibiotici)

Metilprednisolone 80 mg (un corticosteroide per ridurre l'infiammazione)

Pantoprazolo (un farmaco per proteggere lo stomaco)

Insulina (per controllare il diabete)

Salbutamolo/ipratropio bromuro (farmaci per aiutare a respirare)

Enoxaparina (un anticoagulante per prevenire coaguli di sangue)

Ventilazione non invasiva (un trattamento per aiutare a respirare, con diversi parametri impostati e aggiustati)

La signora Rossi è stata ricoverata in un'area specializzata per problemi polmonari per ricevere le cure necessarie. Il suo ricovero è durato dal 21 aprile al 2 maggio. Al suo arrivo in reparto, ha avuto difficoltà a respirare mentre parlava e riceveva il 50% di ossigeno attraverso una maschera. Un'ecografia ha mostrato che i suoi polmoni si muovevano normalmente e che il cuore aveva un aspetto sano. Un esame delle vene nelle sue gambe ha confermato che non c'erano problemi di circolazione sanguigna.

Durante il ricovero, i medici hanno esaminato la quantità di ossigeno e anidride carbonica nel sangue (EGA) della signora Rossi, riscontrando una moderata carenza di ossigeno e una lieve presenza di anidride carbonica. Per migliorare la situazione, è stato impostato un dispositivo che fornisce ossigeno ad alto flusso.

Durante il ricovero, ha ricevuto antibiotici, inizialmente amoxicillina/acido clavulanico e successivamente Bactrim dopo la scoperta di un'infezione da Haemophilus influenzae.

La sig.ra ROSSI ha anche ricevuto trattamenti di fisioterapia per migliorare la sua mobilità.

Alla dimissione, è in buone condizioni generali e stabile. La diagnosi è di insufficienza respiratoria acuta su cronica in paziente con BPCO, con infezione aggiunta (Haemophilus influenzae trovato nell'espettorato).

Appuntamenti futuri: visita di controllo in Pneumologia il 5 luglio 2023 alle 11:45.

Terapia consigliata:

Le prescrizioni seguenti sono suggerimenti per il medico curante, che può apportare le modifiche ritenute opportune.

Beclometasone + formoterolo: 2 dosi al mattino e alla sera (continuazione della terapia domiciliare)

Pantoprazolo: 1 compressa al mattino (nuova prescrizione iniziata durante il ricovero)

Tiotropio bromuro: 2 dosi al mattino (nuova prescrizione iniziata al domicilio)

Simvastatina: 1 compressa al mattino (nuova prescrizione iniziata al domicilio)

Sulfametoxazolo + trimetoprim (Bactrim): 1 compressa a colazione e cena per 3 giorni (nuova prescrizione iniziata durante il ricovero)

Metformina: 1 compressa a cena (nuova prescrizione iniziata al domicilio)

**STUDY 2 – HEALTHCARE STUDENTS**

**CONTROL LETTER– Study 2**

Decorso

Egregio Collega,

si dimette la Sig.ra MARIA ROSSI, nata il 01/01/1950, ricoverata presso la Nostra Struttura in data 17/04/2023.

Allergia a levofloxacina (shock anafilattico con Tavanic), ceftriaxone (orticaria con Rocefin)

Anamnesi familiare e fisiologica: vive con il figlio. Ex fumo (stop Novembre 2020), 10 sig/die per circa 50 anni, non esposizioni professionali (insegnante), autocateterismi urinari, vaccinata per sars-cov2 (5 dosi) ed influenza

Anamnesi patologica remota

-Diabete mellito in terapia con metformina

-Ipercolesterolemia

-Storia di bronchiti ricorrenti

-BOOP noto dal 2012 (eseguita FBS+istologico) in FU c/o pneumologia ospedale Molinette. Esami immunologici 2014 negativi. PFR 2018: alterazione ostruttiva di grado lieve; FVC 2.26 (96%); FEV1 1.7 (92%); IT 66 (79%), TLC 3.8 (88%); RV 1.15 (65%), DLCO 97% 2021: nei limiti di norma; FVC 2.23 (97%), FEV1 1.66 (87%), IT 75, DLCO 97%. Saturimetria notturna in AA (2019): ODI 2.23, CT90 55%. EGA arteriosa in AA (05/2021): pH 7.43, pCO2 36, pO2 66, HCO3-24, COHb 5.4 BPCO in FU c/o pneumologia ospedale Molinette. Ultima spirometria (02/2023 durante ricovero al Mauriziano per IR tipo 1): FEV1 56% in condizioni basali. Risposta significativa al test di broncodilatazione farmacologica (+200 cc + 13% FEV1, + 400 cc + 26% FVC). Ultima visita pneumologica (Mauriziano 02/2023): emitoraci ipomobili, respiro aspro diffusamente ridotto, non rumori aggiunti, prosegue terapia in corso, controllo con PFR tra sei mesi circa

-Ricovero per emoftoe con riscontro di polmonite c/o Molinette (05/2022). TC torace con mdc: consolidazione parenchimale lingula, base sin e paramediastinica, addensamento polmonare apicale del LIS, trattata con steroide e antibiotico.

-Ecocardiogramma 20/02/2023: EF 68% TAPSE 23 mm PAPs 23 mmHg

-Isolato Haemophilus influenzae su escreato resistente ad ampicillina (01/2023), non noto se sottoposto a trattamento

-Isterectomia per prolasso uterino IV stadio con permanenza di alterato svuotamento vescicale per cui la paziente si sottopone ad autocateterismi

Terapia domiciliare: trimbow mattina e sera, simvastatina 40 mg; metformina 500 x 2, O2 notturno 1.5L/min.

Anamnesi patologica prossima

Dal 14/04 insorgenza di febbricola, astenia e dispnea ingravescente. Nega tosse/dolore toracico/cardiopalmo. Non nausea/vomito/diarrea/disuria. Vigile, orientata e collaborante

PAO FC SAT O2 85% in aa tachipnoica ++ EOC: toni cardiaci validi ritmici EOP: MV diffusamente ridotto, broncospasmo ++++ EOA: addome trattabile, non dolente nè dolorabile, peristalsi presente

Non edemi declivi. EGA art in FiO2 28%: pH 7.43, pCO2 44, pO2 53, HCO3 30, lattati 1,6, P/F 190, Na 140, K 3,8 ECO: non versamento pleurico bilateralmente, non linee B bilateralmente, non versamento epicardico, ventricolo sin in limiti, destro non dilatato, VCI in limiti

In PS eseguiti:

-Ematici: WBC 13500/mcl, PCR 41.4, procalcitonina 0.02, creatinina 0.51 mg/dl, AST 20, ALT 16

-RX torace: non addensamenti parenchimali a focolaio. Rinforzo trama interstiziale in sede medio-basale bilaterale. Seni costo-frenici liberi. Ombra cardio-vascolare nei limiti. Calcificazioni lamellari delle pareti dell'arco aortico

- TC torace (19/04): Rispetto al controllo si osserva la pressoché completa detersione dei multipli minuti addensamenti parenchimali con aspetto "ad albero in fiore" precedentemente segnalati nel lobo superiore destro, mentre si osserva la comparsa di una moltitudine di ulteriori analoghi reperti di dimensioni lievemente maggiori nei lobi inferiori, in particolare nelle piramidi basali, e nella lingula, sempre per flogosi delle piccole vie aeree. Ispessimento delle pareti bronchiali in particolare nelle piramidi basali da verosimile flogosi cronica. Parziale riespansione della banda di atelettasia paramediastinica nel lobo medio; sostanzialmente invariato l'analogo reperto nella lingula. Non comparso versamento pleurico. Non versamento pericardico. Leggera riduzione dimensionale di alcuni dei multipli linfonodi presenti in mediastino maggiori in sede pre e sottocarenale con asse corto apparentemente sino a circa 1 cm.

In PS avviata terapia con amoxicillina/clavulanato, azitromicina, metilprednisolone 80 mg, pantoprazolo, insulina, salbutamolo/ipretropio bromuro, enoxaparina a dosaggio di profilassi e NIV, FR 26, FiO2 41, EPAP 5, IPAP 15 con successiva titolazione

Ricovero in Pneumologia area semintensiva per le cure del caso.

Ricovero in Pneumologia Semintensiva/degenza ordinaria dal 21/04 al 02/05

All'ingresso in reparto paziente VCO, tachipnoica all'eloquio in VM 50%. All'ECO: sliding presente bilateralmente. Pattern A diffuso. FE iespettiva conservata, camere dx non dilatate, VCI 1.39 collabente > 50%

CUS: asse femoro popliteo comprimibile bilateralmente

Eseguito EGA in MV 50%; ipossiemia moderata, ipercapnia lieve. Si impostavano HFNC 60/0.6.

Durante la degenza si è assistito a un progressivo miglioramento degli scambi respiratori con svezzamento dall'ossigeno terapia ad alti flussi e mantenimento di O2 a bassi flussi. Al momento della dimissione paziente eupnoica in CN 1 1/min (già in possesso di O2 domiciliare).

È stata proseguita la terapia antibiotica con amoxi/ac. clavulanico dal 21/04 al 28/04. In seguito al riscontro di positività su escreato a Haemophilus influenzae (Carica microbica: 1.000.000 UFC./ml) è stata sostituita con Bactrim per OS (dal 28/04).

La paziente è stata presa in carico dal punto di vista fisioterapico, con ripresa della mobilizzazione in autonomia.

Si dimette la paziente in data odierna, in buone condizioni generali. Parametri vitali stabili

Diagnosi alla dimissione IR acuta su cronica in BPCO riacutizzata con sovrapposizione flogistica (es batteriologico positivo su espettorato per H. Influenzae)

Prossimi appuntamenti: -Visita pneumologica di controllo in data 5/7/2023 h 11.45

Terapia consigliata:

La terapia consigliata è una indicazione per il Suo medico curante, che potrebbe apportarvi le modifiche ritenute opportune (prodotti equivalenti o similari sulla base delle limitazioni di prescrivibilità previste dalle note CUF o della rimborsabilità da parte del SSN): alcuni farmaci indicati potrebbero essere soggetti a pagamento.

| Farmaco prescritto | Posologia | Annotazioni medico | Stato prescrizione | Fascia/nota AIFA |
| --- | --- | --- | --- | --- |
| Mattino | | | | |
| Beclometasone + formoterolo 100/6 mcg per erogaz. Soluz. Pressurizzata | 2 DOS |  | Continuazione terapia domiciliare | A |
| Pantoprazolo sale sodico 20 mg 28 cpr gastroresistenti | 1 CPR |  | Nuova prescrizione iniziata in ricovero | A nota 48 |
| Tiotropio bromuro 2,5 mcg soluz per inalazione respirat + 1 cartuccia da 60 erogazioni | 2 DOS |  | Nuova prescrizione iniziata al domicilio | A |
| Simavastatina | 1 CPR |  | Nuova prescrizione iniziata al domicilio | A Nota 13 |
| Colazione | | | | |
| Sulfametoxazolo + trimetoprim 160 mg + 800 mg 16 compresse | 1 CPR | Assumere per 3 gg | Nuova prescrizione iniziata in ricovero | A |
| Cena | | | | |
| Sulfametoxazolo + trimetoprim 160 mg + 800 mg 16 compresse | 1 CPR | Assumere per 3 gg | Nuova prescrizione iniziata in ricovero | A |
| Metformina cloridrato 500 mg 30 cpr rivestite | 1 CPR |  | Nuova prescrizione iniziata al domicilio | A |
| Sera | | | | |
| Beclometasone + formoterolo 100/6 mcg per erogaz. Soluz. Pressurizzata | 2 DOS |  | Continuazione terapia domiciliare | A |

**ARTIFICIAL INTELLIGENCE LETTER– Study 2**

Gentile Collega,

la signora Maria Rossi, nata il 01/01/1950, è stata dimessa dal nostro ospedale dopo il suo ricovero iniziato il 17/04/2023.

La signora Rossi è allergica a due farmaci: levofloxacina (ha avuto una reazione grave chiamata shock anafilattico) e ceftriaxone (ha avuto un'eruzione cutanea chiamata orticaria).

Maria vive con suo figlio. Ha smesso di fumare nel novembre 2020 dopo aver fumato 10 sigarette al giorno per circa 50 anni. Ha lavorato come insegnante, quindi non ha avuto esposizioni professionali nocive. Pratica l'autocateterismo urinario (un metodo per svuotare la vescica quando si hanno difficoltà a farlo normalmente). Ha ricevuto 5 dosi di vaccino contro il COVID-19 e il vaccino contro l'influenza.

Tra le sue condizioni mediche passate ci sono:

- Diabete, per il quale assume metformina
- Colesterolo alto
- Bronchiti ricorrenti
- Una malattia polmonare chiamata BOOP, diagnosticata nel 2012, per la quale è seguita dall'ospedale Molinette
- Ricovero per sanguinamento polmonare e polmonite nel 2022, trattata con steroidi e antibiotici
- Ecocardiogramma nel 2023 con risultati generalmente normali
- Un batterio chiamato Haemophilus influenzae resistente all'ampicillina è stato trovato nel suo espettorato nel gennaio 2023, ma non si sa se è stata trattata per questo
- Isterectomia (rimozione dell'utero) per prolasso uterino di quarto grado, con difficoltà persistenti nello svuotamento della vescica, per le quali pratica l'autocateterismo.

La terapia che la sig.ra ROSSI segue a casa include: Trimbow (un farmaco per il respiro) al mattino e alla sera, simvastatina 40 mg (per il colesterolo alto), metformina 500 mg due volte al giorno (per il diabete) e ossigeno supplementare di notte a 1,5 L/min.

Condizioni mediche recenti

Dal 14 aprile, la sig.ra ROSSI ha avuto febbre leggera, stanchezza e difficoltà respiratorie che peggiorano. Non ha avuto tosse, dolore al petto, palpitazioni, nausea, vomito, diarrea o difficoltà a urinare. Durante la visita, era sveglia, orientata e collaborativa. La sua ossigenazione era bassa (85%) e respirava rapidamente.

Gli esami fatti in Pronto Soccorso includono:

- Esami del sangue: aumento dei globuli bianchi (indicativo di un'infezione), proteina C-reattiva alta (indicativa di infiammazione), livelli normali di procalcitonina, creatinina, enzimi epatici (AST e ALT)
- Radiografia al torace: nessun segno di infezione polmonare, ma un rinforzo della struttura polmonare nelle zone medie e basali dei polmoni
- Tomografia computerizzata (TC) al torace: rispetto all'esame precedente, alcuni segni di infiammazione nelle piccole vie aeree sono scomparsi, mentre ne sono comparsi altri in diverse aree dei polmoni, soprattutto nelle zone basali e nella lingula (una parte del polmone sinistro). I linfonodi nel mediastino (la parte centrale del torace) sono leggermente ridotti di dimensioni.

In Pronto Soccorso, la sig.ra ROSSI ha iniziato un trattamento con:

- Amoxicillina/clavulanato e azitromicina (antibiotici)
- Metilprednisolone 80 mg (un corticosteroide per ridurre l'infiammazione)
- Pantoprazolo (un farmaco per proteggere lo stomaco)
- Insulina (per controllare il diabete)
- Salbutamolo/ipratropio bromuro (farmaci per aiutare a respirare)
- Enoxaparina (un anticoagulante per prevenire coaguli di sangue)
- Ventilazione non invasiva (un trattamento per aiutare a respirare, con diversi parametri impostati e aggiustati)

La signora Rossi è stata ricoverata in un'area specializzata per problemi polmonari per ricevere le cure necessarie. Il suo ricovero è durato dal 21 aprile al 2 maggio. Al suo arrivo in reparto, ha avuto difficoltà a respirare mentre parlava e riceveva il 50% di ossigeno attraverso una maschera. Un'ecografia ha mostrato che i suoi polmoni si muovevano normalmente e che il cuore aveva un aspetto sano. Un esame delle vene nelle sue gambe ha confermato che non c'erano problemi di circolazione sanguigna.

Durante il ricovero, i medici hanno esaminato la quantità di ossigeno e anidride carbonica nel sangue (EGA) della signora Rossi, riscontrando una moderata carenza di ossigeno e una lieve presenza di anidride carbonica. Per migliorare la situazione, è stato impostato un dispositivo che fornisce ossigeno ad alto flusso.

Durante il ricovero, ha ricevuto antibiotici, inizialmente amoxicillina/acido clavulanico e successivamente Bactrim dopo la scoperta di un'infezione da Haemophilus influenzae.

La sig.ra ROSSI ha anche ricevuto trattamenti di fisioterapia per migliorare la sua mobilità.

Alla dimissione, è in buone condizioni generali e stabile. La diagnosi è di insufficienza respiratoria acuta su cronica in paziente con BPCO, con infezione aggiunta (Haemophilus influenzae trovato nell'espettorato).

Appuntamenti futuri: visita di controllo in Pneumologia il 5 luglio 2023 alle 11:45.

Terapia consigliata:

Le prescrizioni seguenti sono suggerimenti per il medico curante, che può apportare le modifiche ritenute opportune.

- Beclometasone + formoterolo: 2 dosi al mattino e alla sera (continuazione della terapia domiciliare)
- Pantoprazolo: 1 compressa al mattino (nuova prescrizione iniziata durante il ricovero)
- Tiotropio bromuro: 2 dosi al mattino (nuova prescrizione iniziata al domicilio)
- Simvastatina: 1 compressa al mattino (nuova prescrizione iniziata al domicilio)
- Sulfametoxazolo + trimetoprim (Bactrim): 1 compressa a colazione e cena per 3 giorni (nuova prescrizione iniziata durante il ricovero)
- Metformina: 1 compressa a cena (nuova prescrizione iniziata al domicilio)

Nota: alcune delle prescrizioni potrebbero essere soggette a pagamento.

**M2.** Clinical content-fidelity assessment

**Methods**

Two study authors, both medical doctors, independently compared each AI-generated letter with the corresponding original letter. The assessment focused on the information domains evaluated in the participant comprehension questionnaire: main diagnosis, discharge medications, antibiotic instructions, diagnostic tests, follow-up instructions, and, for Study 2, the chronological sequence of clinical events. Each domain was assessed for clinical accuracy and completeness using a three-point scale (0 = inaccurate/incomplete, 1 = partially accurate/complete, and 2 = fully accurate/complete). The presence of hallucinated, fabricated, or additional information was assessed for each domain and globally for each letter (0 = absent, 1 = present).

Inter-rater agreement was evaluated using percentage agreement and Cohen’s kappa. When both raters assigned the same category to all items, Cohen’s kappa could not be estimated because there was no variability in the ratings.

**Results**

For clinical accuracy, the raters agreed on 9 of 11 domains (81.8%). Cohen’s kappa was 0.00 because the observed agreement was equal to the high agreement expected from the markedly unbalanced marginal distributions. The two disagreements concerned the description of diagnostic tests, which one rater considered fully accurate and the other partially accurate.

For completeness, agreement was observed for 10 of 11 domains (90.9%), with a Cohen’s kappa of 0.62 (standard error 0.28). The only disagreement concerned the completeness of the diagnostic-test information in Study 1.

Neither rater identified hallucinated, fabricated, or additional information in any of the 13 assessments, resulting in 100% agreement. Cohen’s kappa could not be estimated because both raters assigned the same category to all assessments.

**M3.** Quantitative readability and document length

Readability was assessed retrospectively using the Gulpease index,^2^ a measure specifically developed for Italian-language texts. The index was calculated as (89 + [(300*S) - (10*L)]/W), where (S) is the number of sentences, (L) the number of letters, and (W) the number of words; higher scores indicate greater readability. Document length was assessed using word count. For documents containing lists or tables, each standalone item conveying a complete clinical statement or instruction was treated as a sentence-equivalent unit, irrespective of terminal punctuation. Headings, section labels, abbreviations, and decimal points were not considered sentence boundaries. The same criteria were applied to the original and AI-generated letters.

In Study 1, the AI-generated letter was shorter than the original letter (365 vs 388 words; 5.9% reduction), while the Gulpease indices were similar (48.6 vs 49.9, respectively). In Study 2, the AI-generated letter was also shorter (814 vs 1,024 words; 20.5% reduction), with comparable Gulpease indices (49.1 vs 48.7). Thus, the AI-generated letters were consistently shorter, whereas no consistent improvement in formula-based readability was observed.

| **Study** | **Letter** | **Sentence-equivalent units** | **Letters** | **Words** | **Gulpease index** |
| --- | --- | --- | --- | --- | --- |
| Study 1 | Original | 26 | 2,298 | 388 | 49.9 |
| Study 1 | AI-generated | 26 | 2,254 | 365 | 48.6 |
| Study 2 | Original | 68 | 6,171 | 1,024 | 48.7 |
| Study 2 | AI-generated | 51 | 4,776 | 814 | 49.1 |

**M4.** Comprehension assessment

The study-specific comprehension items were reviewed before administration by four medical doctors; however, no formal pilot testing or quantitative psychometric validation was conducted. Comprehension was evaluated across five core domains: (1) identification of the main diagnosis; (2) recognition of prescribed medications; (3) understanding of antibiotic instructions, including dosage, frequency, and duration; (4) identification of diagnostic tests performed during hospitalisation; and (5) identification of the scheduled follow-up appointment.

To facilitate online completion, Study 1 predominantly used structured-response items, while the diagnosis item was open-ended. Each correctly answered domain received one point, yielding a total score ranging from 0 to 5. In Study 2, the diagnosis item remained open-ended, the medication and diagnostic-test items were converted to open-ended format, and an additional chronological-sequencing item was introduced to increase assessment depth and reduce guessing. Consequently, the Study 2 score ranged from 0 to 6. Scores were analysed separately, and no direct cross-study comparison was intended.

All responses were classified as correct or incorrect according to predefined criteria. Structured-response items were scored against predefined correct answers, while open-ended responses were independently assessed by two study authors, with disagreements resolved through discussion involving the full research team. Assessors were not blinded to study allocation, and inter-rater agreement was not quantified.

## **M5.** Secondary outcome measures

Participants rated their agreement with a series of statements addressing: ease of reading and understanding; organization of information; clarity in addressing doubts and concerns; presence of complex or unclear terminology; ability to follow post-discharge instructions without additional explanation; overall satisfaction; and preference for a more easily understandable version of the discharge letter.

## **M6.** Residual normality check

Residual normality was assessed separately for each study by visually comparing kernel density estimates of the residuals with the corresponding normal density curves. The distributions were approximately symmetric and showed no substantial departures from normality.

**Study 1**

**Study 2**

**References:**

1.	García-Olmos L, Alberquilla A, Ayala V, et al. Comorbidity in patients with chronic obstructive pulmonary disease in family practice: a cross sectional study. BMC Fam Pract. 2013;14:11. doi:10.1186/1471-2296-14-11

2.	Lucisano P, Piemontese ME. GULPEASE: una formula per la predizione della difficoltà dei testi in lingua italiana. Scuola e città. 1988;3(31):110–24.

# SUPPLEMENTARY FIGURES

**Fig. S1.** Flow diagram of the recruitment process (Study 1)

## **Fig. S2.** Flow diagram of the recruitment process (Study 2)

# SUPPLEMENTARY TABLES

## **Table SI** Baseline socio-demographic characteristics and health literacy scores stratified by experimental and control groups (Study 1)

|  | **Original letter**  **(n=166)**  **N (%)** | **AI-generated letter**  **(n=175)**  **N (%)** | **χ2 Test**  **p-value** |
| --- | --- | --- | --- |
| **Age (years)*** | 42 (29-56) | 45 (29-59) | 0.248 |
| **Gender** |  |  | 0.505 |
| Female | 81 (48.8) | 82 (46.9) |  |
| Male | 83 (50) | 93 (53.1) |  |
| Other/prefer not to answer | 2 (0.2) | 0 |  |
| **Nationality** |  |  | 0.278 |
| Other | 11 (6.6) | 7 (4) |  |
| Italian | 155 (93.4) | 168 (96) |  |
| **Mother tongue** |  |  | 0.408 |
| Other | 11 (6.6) | 8 (4.6) |  |
| Italian | 155 (93.4) | 167 (95.4) |  |
| **Education level** |  |  | 0.713 |
| Middle school or below | 12 (7.2) | 10 (5.7) |  |
| High school diploma | 67 (40.4) | 64 (36.6) |  |
| Bachelor’s degree | 31 (18.7) | 40 (22.9) |  |
| Master’s degree or above | 56 (34.7) | 61 (34.8) |  |
| **Working, studying or have worked as a healthcare professional** |  |  | **0.041** |
| Yes | 41 (24.7) | 61 (34.8) |  |
| No | 125 (75.3) | 114 (65.2) |  |
| **Perceived economic situation** |  |  | 0.976 |
| Excellent or Good | 144 (86.7) | 151(86.2) |  |
| Poor or Insufficient | 22 (13.3) | 24 (13.8) |  |
| **Perceived health status** |  |  | 0.939 |
| Excellent or Good | 139 (83.7) | 146 (83.4) |  |
| Poor or Insufficient | 27 (16.3) | 29 (16.6) |  |
| **Healthcare services usage** |  |  | 0.345 |
| Users (at least 1-2 times per year) | 42 (25.3) | 50 (28.6) |  |
| Non-users (less than 1 times per year) | 124 (74.7) | 125 (71.4) |  |
| **Health literacy score** |  |  | 0.809 |
| Inadequate | 37 (22.3) | 34 (19.4) |  |
| Problematic | 119 (71.7) | 130 (74.3) |  |
| Sufficient | 10 (6.0) | 11 (6.3) |  |

*Age: Data are reported as median (IQR) and p-value is obtained via Mann-Whitney U test.

## **Table SII** Differences in items composing the primary outcome between intervention and control groups (Study 1)

| **Primary outcome item** | **Arm of the study** | **Correct answer N (%)** | **Incorrect answer N (%)** | **Odds Ratio** | **95% CI** | **P-value** |
| --- | --- | --- | --- | --- | --- | --- |
| **Recognition of the Main Diagnosis** |  |  |  |  |  |  |
|  | Original letter | 79 (47.6%) | 87 (52.4%) | Ref. |  |  |
|  | AI-generated letter | 147 (84.0%) | 28 (16.0%) | 5.78 | 3.49 – 9.59 | **<0.001** |
| **Identification of Medications Prescribed at Discharge** |  |  |  |  |  |  |
|  | Original letter | 14 (8.4%) | 152 (91.6%) | Ref. |  |  |
|  | AI-generated letter | 102 (58.3%) | 73 (41.7%) | 14.8 | 7.94 – 27.7 | **<0.001** |
| **Understanding of Antibiotic Administration Instructions (Dosage, Frequency, Duration)** |  |  |  |  |  |  |
|  | Original letter | 37 (22.3%) | 129 (77.7%) | Ref. |  |  |
|  | AI-generated letter | 127 (72.6%) | 48 (27.4%) | 9.22 | 5.63 – 15.1 | **<0.001** |
| **Listing of Diagnostic Exams Performed During the Hospital Stay in Pulmonology Department** |  |  |  |  |  |  |
|  | Original letter | 15 (9.0%) | 151 (91%) | Ref. |  |  |
|  | AI-generated letter | 27 (15.2%) | 148 (84.8%) | 2.70 | 1.42 – 5.13 | **0.002** |
| **Identification of Scheduled Follow-Up Appointments** |  |  |  |  |  |  |
|  | Original letter | 151 (91.0%) | 15 (9.0%) | Ref. |  |  |
|  | AI-generated letter | 166 (93.0%) | 9 (5.1%) | 1.83 | 0.779 – 4.31 | 0.160 |

Correct and incorrect answers are reported as N (%). Chi-squared tests are reported with odds ratio, CI 95% and p-value

## **Table SIII** Multiple Linear Regression Model of Intervention group – AI-generated version of the discharge letter. Dependent variable: comprehension score (Study 1)

|  | **Adjusted Estimate** | **Standard Error** | **Lower 95% CI** | **Upper 95% CI** | **p-value** |
| --- | --- | --- | --- | --- | --- |
| **Age (years)** | 0.013 | 0.007 | -6.84e−4 | 0.026 | 0.063 |
| **Gender** |  |  |  |  |  |
| Female | Ref. |  |  |  |  |
| Male | 0.750 | 0.190 | 0.374 | 1.124 | **<0.001** |
| **Mother tongue** |  |  |  |  |  |
| Italian | Ref. |  |  |  |  |
| Other | 0.276 | 0.441 | -0.596 | 1.147 | 0.533 |
| **Education level** |  |  |  |  |  |
| Middle school or below | Ref. |  |  |  |  |
| High school diploma | 0.715 | 0.397 | -0.068 | 1.499 | 0.073 |
| Bachelor’s degree | 1.158 | 0.417 | 0.334 | 1.982 | **0.006** |
| Master’s degree or above | 0.884 | 0.423 | 0.048 | 1.720 | **0.038** |
| **Working, studying or have worked as a healthcare professional** |  |  |  |  |  |
| No | Ref. |  |  |  |  |
| Yes | 0.415 | 0.199 | 0.022 | 0.807 | **0.039** |
| **Perceived economic situation** |  |  |  |  |  |
| Poor or Insufficient | Ref. |  |  |  |  |
| Excellent or Good | -0.343 | 0.539 | -0.882 | 0.196 | 0.211 |
| **Perceived health status** |  |  |  |  |  |
| Poor or Insufficient | Ref. |  |  |  |  |
| Excellent or Good | 0.356 | 0.251 | 0.105 | 0.608 | **0.006** |
| **Healthcare services usage** |  |  |  |  |  |
| Non-users (less than 1 times per year) | Ref. |  |  |  |  |
| Users (at least 1-2 times per year) | 0.042 | 0.196 | -0.345 | 0.430 | 0.829 |
| **Health literacy score** |  |  |  |  |  |
| Inadequate | Ref. |  |  |  |  |
| Problematic | 0.438 | 0.226 | -0.009 | 0.884 | 0.055 |
| Sufficient | 1.023 | 0.407 | 0.220 | 1.827 | **0.013** |

## **Table SIV** Multiple Linear Regression Model of Control group – Original version of the discharge letter. Dependent variable: comprehension score (Study 1)

|  | **Adjusted Estimate** | **Standard Error** | **Lower 95% CI** | **Upper 95% CI** | **p-value** |
| --- | --- | --- | --- | --- | --- |
| **Age (years)** | 0.000157 | 0.00473 | 0.00951 | 0.00919 | 0.974 |
| **Gender** |  |  |  |  |  |
| Female | Ref. |  |  |  |  |
| Male | -0.0728 | 0.146 | -0.361 | 0.215 | 0.619 |
| **Mother tongue** |  |  |  |  |  |
| Italian | Ref. |  |  |  |  |
| Other | -0.887 | 0.301 | -1.482 | -0.291 | **0.004** |
| **Education level** |  |  |  |  |  |
| Middle school or below | Ref. |  |  |  |  |
| High school diploma | -0.00461 | 0.281 | -0.559 | 0.550 | 0.987 |
| Bachelor’s degree | 0.194 | 0.312 | -0.423 | 0.811 | 0.536 |
| Master’s degree or above | 0.376 | 0.304 | -0.226 | 0.977 | 0.219 |
| **Working, studying or have worked as a healthcare professional** |  |  |  |  |  |
| No | Ref. |  |  |  |  |
| Yes | 1.214 | 0.163 | 0.891 | 1.536 | **< 0.001** |
| **Perceived economic situation** |  |  |  |  |  |
| Poor or Insufficient | Ref. |  |  |  |  |
| Excellent or Good | -0.114 | 0.425 | -0.539 | 0.311 | 0.596 |
| **Perceived health status** |  |  |  |  |  |
| Poor or Insufficient | Ref. |  |  |  |  |
| Excellent or Good | 0.0848 | 0.167 | -0.0827 | 0.252 | 0.319 |
| **Healthcare services usage** |  |  |  |  |  |
| Non-users (less than 1 times per year) | Ref. |  |  |  |  |
| Users (at least 1-2 times per year) | 0.0223 | 0.153 | -0.279 | 0.324 | 0.884 |
| **Health literacy score** |  |  |  |  |  |
| Inadequate | Ref. |  |  |  |  |
| Problematic | -0.0238 | 0.169 | -0.355 | 0.307 | 0.887 |
| Sufficient | 0.755 | 0.305 | 0.151 | 1.359 | **0.015** |

## **Table SV** Multiple linear regression model for the primary outcome (comprehension score), Subsample of non-healthcare professionals. (Study 1)

|  | **Adjusted Estimate** | **Standard Error** | **Lower 95% CI** | **Upper 95% CI** | **P value** |
| --- | --- | --- | --- | --- | --- |
| **Group** |  |  |  |  |  |
| Control group |  |  |  |  |  |
| Intervention group | 1.667 | 0.134 | 1.402 | 1.932 | <0.001 |
| **Age (years)** | 0.009 | 0.004 | 0.000 | 0.017 | 0.057 |
| **Gender** |  |  |  |  |  |
| Female |  |  |  |  |  |
| Male | 0.430 | 0.151 | 0.132 | 0.727 | 0.005 |
| **Mother tongue** |  |  |  |  |  |
| Italian |  |  |  |  |  |
| Other | -0.058 | 0.296 | -0.641 | 0.526 | 0.846 |
| **Education level** |  |  |  |  |  |
| Middle school or below |  |  |  |  |  |
| High school diploma | 0.393 | 0.273 | -0.145 | 0.931 | 0.151 |
| Bachelor’s degree | 0.934 | 0.312 | 0.319 | 1.548 | 0.003 |
| Master’s degree or above | 0.809 | 0.306 | 0.205 | 1.412 | 0.009 |
| **Perceived economic situation** |  |  |  |  |  |
| Poor or Insufficient |  |  |  |  |  |
| Excellent or Good | -0.149 | 0.204 | -0.551 | 0.253 | 0.465 |
| **Perceived health status** |  |  |  |  |  |
| Poor or Insufficient |  |  |  |  |  |
| Excellent or Good | 0.204 | 0.198 | -0.185 | 0.594 | 0.302 |
| **Healthcare services usage** |  |  |  |  |  |
| Non-users (less than 1 times per year) |  |  |  |  |  |
| Users (at least 1-2 times per year) | 0.148 | 0.159 | -0.165 | 0.461 | 0.353 |
| **Health literacy score** |  |  |  |  |  |
| Inadequate |  |  |  |  |  |
| Problematic | 0.272 | 0.178 | -0.079 | 0.623 | 0.128 |
| Sufficient | 1.222 | 0.323 | 0.585 | 1.859 | <0.001 |

## **Table SVI** Multiple linear regression model for the primary outcome (comprehension score), Subsample of healthcare professionals. (Study 1)

|  | **Adjusted Estimate** | **Standard Error** | **Lower 95% CI** | **Upper 95% CI** | **P value** |
| --- | --- | --- | --- | --- | --- |
| **Group** |  |  |  |  |  |
| Control group |  |  |  |  |  |
| Intervention group | 0.458 | 0.218 | 0.026 | 0.891 | 0.038 |
| **Age (years)** | -0.012 | 0.010 | -0.031 | 0.007 | 0.219 |
| **Gender** |  |  |  |  |  |
| Female |  |  |  |  |  |
| Male | 0.391 | 0.227 | -0.060 | 0.841 | 0.088 |
| **Mother tongue** |  |  |  |  |  |
| Italian |  |  |  |  |  |
| Other | -1.541 | 1.093 | -3.713 | 0.631 | 0.162 |
| **Education level** |  |  |  |  |  |
| Middle school or below |  |  |  |  |  |
| High school diploma | 0.902 | 1.086 | -1.255 | 3.059 | 0.408 |
| Bachelor’s degree | 1.063 | 1.083 | -1.089 | 3.215 | 0.329 |
| Master’s degree or above | 1.287 | 1.093 | -0.884 | 3.458 | 0.242 |
| **Perceived economic situation** |  |  |  |  |  |
| Poor or Insufficient |  |  |  |  |  |
| Excellent or Good | -0.415 | 0.414 | -1.238 | 0.408 | 0.319 |
| **Perceived health status** |  |  |  |  |  |
| Poor or Insufficient |  |  |  |  |  |
| Excellent or Good | 0.954 | 0.379 | 0.201 | 1.706 | 0.014 |
| **Healthcare services usage** |  |  |  |  |  |
| Non-users (less than 1 times per year) |  |  |  |  |  |
| Users (at least 1-2 times per year) | 0.102 | 0.218 | -0.330 | 0.534 | 0.641 |
| **Health literacy score** |  |  |  |  |  |
| Inadequate |  |  |  |  |  |
| Problematic | -0.101 | 0.271 | -0.640 | 0.438 | 0.710 |
| Sufficient | 0.203 | 0.472 | -0.736 | 1.141 | 0.669 |

## **Table SVII** Baseline socio-demographic characteristics and health literacy scores stratified by experimental and control groups (Study 2)

|  | **Original letter**  **(n=407)**  **N (%)** | **AI-generated letter (n=384)**  **N (%)** | **χ2 Test**  **p-value** |
| --- | --- | --- | --- |
| **Age (years)*** | 20.72 (19-21) | 20.72 (19-21) |  |
| **Gender** |  |  | 0.455 |
| Female | 306 (75.2) | 279 (72.6) |  |
| Male | 99 (24.3) | 102 (26.6) |  |
| Other | 2 (0.5) | 3 (0.8) |  |
| **Nationality** |  |  | 0.289 |
| Italian | 393 (96.6) | 365 (95.1) |  |
| Other | 14 (3.4) | 19 (4.9) |  |
| **Mother tongue** |  |  | 0.871 |
| Italian | 388 (95.3) | 367 (95.6) |  |
| Other (prefer not to answer) | 19 (4.7) | 17 (4.4) |  |
| **Degree course** |  |  | 0.309 |
| Medicine and Surgery | 210 (51.6) | 212 (55.2) |  |
| Nursing | 197 (48.4) | 172 (44.8) |  |
| **Year of study** |  |  | 0.375 |
| 1° | 200 (49.1) | 207 (53.9) |  |
| 2° | 136 (33.4) | 110 (28.6) |  |
| 3° | 69 (17.0) | 67 (17.4) |  |
| 4° | 1 (0.2) | 0 (0.0) |  |
| 5° | 1 (0.2) | 0 (0.0) |  |
| **Perceived economic situation** |  |  |  |
| Excellent or Good | 361 (88.7) | 340 (88.5) | 0.945 |
| Poor or Insufficient | 46 (11.3) | 44 (11.5) |  |
| **Perceived health status** |  |  | 0.933 |
| Excellent or Good | 379 (93.1) | 357 (93.0) |  |
| Poor or Insufficient | 28 (6.9) | 27 (7.0) |  |
| **Frequency of use of healthcare services** |  |  | 0.618 |
| Users (at least 1-2 times per year) | 276 (67.8) | 254 (66.1) |  |
| Non-users (less than 1 times per year) | 131 (32.2) | 130 (33.9) |  |
| **Health literacy score** |  |  | 0.106 |
| Inadequate | 36 (8.9) | 22 (5.8) |  |
| Problematic | 332 (82.2) | 314 (82.2) |  |
| Sufficient | 36 (8.9) | 46 (12.0) |  |

## **Table SVIII** Differences in items composing the primary outcome between intervention and control groups (Study 2)

| **Primary outcome item** | **Arm of the study** | **Correct answer N (%)** | **Incorrect answer N (%)** | **Odds Ratio** | **95% CI** | **P-value** |
| --- | --- | --- | --- | --- | --- | --- |
| **Recognition of the Main Diagnosis** |  |  |  |  |  |  |
|  | Original letter | 246 (60.4%) | 161 (39.6%) | Ref. |  |  |
|  | AI-generated letter | 327 (85.2) | 57 (14.8%) | 3.75 | 2.66-5.30 | **<0.001** |
| **Identification of Medications Prescribed at Discharge** |  |  |  |  |  |  |
|  | Original letter | 176 (43.2%) | 231 (56.8%) | Ref. |  |  |
|  | AI-generated letter | 283 (73.7%) | 101 (26.3%) | 3.68 | 2.72-4.96 | **<0.001** |
| **Understanding of Antibiotic Administration Instructions (Dosage, Frequency, Duration)** |  |  |  |  |  |  |
|  | Original letter | 231 (56.8%) | 176 (43.2%) | Ref. |  |  |
|  | AI-generated letter | 264 (68.8%) | 120 (31.2%) | 1.68 | 1.25-2.24 | **0.001** |
| **Listing of Diagnostic Exams Performed During the Hospital Stay in Pulmonology Department** |  |  |  |  |  |  |
|  | Original letter | 122 (30.0%) | 285 (70.0%) | Ref. |  |  |
|  | AI-generated letter | 179 (46.6%) | 205 (53.4%) | 2.03 | 1.52-2.72 | **<0.001** |
| **Reconstruction of the correct chronological sequence of events** |  |  |  |  |  |  |
|  | Original letter | 50 (12.3%) | 357 (87.7%) | Ref. |  |  |
|  | AI-generated letter | 84 (21.9%) | 300 (78.1%) | 2.00 | 1.36-2.93 | **<0.001** |
| **Identification of Scheduled Follow-Up Appointments** |  |  |  |  |  |  |
|  | Original letter | 385 (94.6%) | 22 (5.4%) | Ref. |  |  |
|  | AI-generated letter | 358 (93.2%) | 26 (6.8%) | 0.79 | 0.44-1.41 | 0.422 |

Correct and incorrect answers are reported as N (%). Chi-squared tests are reported with odds ratio, CI 95% and p-value

## **Table SIX** Multiple Linear Regression Model of Intervention group – AI-generated version of the discharge letter. Dependent variable: comprehension score (Study 2)

|  | **Adjusted Estimate** | **Standard Error** | **Lower 95% CI** | **Upper 95% CI** | **p-value** |
| --- | --- | --- | --- | --- | --- |
| **Age (years)** | <0.001 | 0.024 | -0.047 | 0.047 | 0.999 |
| **Gender** |  |  |  |  |  |
| Female | Ref. |  |  |  |  |
| Male | -0.256 | 0.138 | -0.527 | 0.015 | 0.064 |
| **Nationality** |  |  |  |  |  |
| Italian | Ref. |  |  |  |  |
| Other | -0.445 | 0.433 | -1.297 | 0.407 | 0.305 |
| **Mother tongue** |  |  |  |  |  |
| Italian | Ref. |  |  |  |  |
| Other | 0.184 | 0.454 | -0.709 | 1.076 | 0.686 |
| **Degree course** |  |  |  |  |  |
| Medicine and surgery | Ref. |  |  |  |  |
| Nursing | -0.609 | 0.126 | -0.856 | -0.361 | **<0.001** |
| **Year of study** |  |  |  |  |  |
| 1° | Ref. |  |  |  |  |
| 2° | 0.087 | 0.144 | -0.196 | 0.370 | 0.546 |
| >3° | 0.694 | 0.175 | 0.349 | 1.039 | **<0.001** |
| **Perceived economic situation** |  |  |  |  |  |
| Poor or Insufficient | Ref. |  |  |  |  |
| Excellent or Good | -0.100 | 0.194 | -0.482 | 0.283 | 0.609 |
| **Perceived health status** |  |  |  |  |  |
| Poor or Insufficient | Ref. |  |  |  |  |
| Excellent or Good | 0.602 | 0.252 | 0.107 | 1.097 | **0.017** |
| **Healthcare services usage** |  |  |  |  |  |
| Non-users (less than 1 times per year) | Ref. |  |  |  |  |
| Users (at least 1-2 times per year) | 0.330 | 0.134 | 0.066 | 0.593 | **0.014** |
| **Health literacy score** |  |  |  |  |  |
| Inadequate | Ref. |  |  |  |  |
| Problematic | -0.103 | 0.274 | -0.641 | 0.435 | 0.708 |
| Sufficient | -0.083 | 0.319 | -0.710 | 0.545 | 0.795 |

## **Table SX** Multiple Linear Regression Model of Control group – Original version of the discharge letter. Dependent variable: comprehension score (Study 2)

|  | **Adjusted Estimate** | **Standard Error** | **Lower 95% CI** | **Upper 95% CI** | **p-value** |
| --- | --- | --- | --- | --- | --- |
| **Age (years)** | 0.005 | 0.017 | -0.028 | 0.038 | 0.757 |
| **Gender** |  |  |  |  |  |
| Female | Ref. |  |  |  |  |
| Male | -0.216 | 0.153 | -0.516 | 0.083 | 0.157 |
| **Nationality** |  |  |  |  |  |
| Italian | Ref. |  |  |  |  |
| Other | 0.167 | 0.589 | -0.991 | 1.325 | 0.777 |
| **Mother tongue** |  |  |  |  |  |
| Italian | Ref. |  |  |  |  |
| Other | -0.367 | 0.524 | -1.397 | 0.663 | 0.484 |
| **Degree course** |  |  |  |  |  |
| Medicine and surgery | Ref. |  |  |  |  |
| Nursing | -0.398 | 0.135 | -0.664 | -0.132 | **0.003** |
| **Year of study** |  |  |  |  |  |
| 1° | Ref. |  |  |  |  |
| 2° | 0.119 | 0.143 | -0.162 | 0.400 | 0.406 |
| >3° | 0.701 | 0.179 | 0.349 | 1.053 | **<0.001** |
| **Perceived economic situation** |  |  |  |  |  |
| Poor or Insufficient | Ref. |  |  |  |  |
| Excellent or Good | -0.120 | 0.214 | -0.541 | 0.302 | 0.577 |
| **Perceived health status** |  |  |  |  |  |
| Poor or Insufficient | Ref. |  |  |  |  |
| Excellent or Good | -0.223 | 0.260 | -0.734 | 0.289 | 0.392 |
| **Healthcare services usage** |  |  |  |  |  |
| Non-users (less than 1 times per year) | Ref. |  |  |  |  |
| Users (at least 1-2 times per year) | 0.111 | 0.136 | -0.155 | 0.378 | 0.412 |
| **Health literacy score** |  |  |  |  |  |
| Inadequate | Ref. |  |  |  |  |
| Problematic | -0.073 | 0.226 | -0.518 | 0.371 | 0.746 |
| Sufficient | 0.021 | 0.304 | -0.577 | 0.618 | 0.946 |

## **Table SXI** Multiple Linear Regression Model of medical students’ subgroup. Dependent variable: comprehension score (Study 2)

|  | **Adjusted Estimate** | **Standard Error** | **Lower 95% CI** | **Upper 95% CI** | **p-value** |
| --- | --- | --- | --- | --- | --- |
| **Group** |  |  |  |  |  |
| Control group | Ref. |  |  |  |  |
| Intervention group | 0.977 | 0.119 | 0.743 | 1.210 | **<0.001** |
| **Age (years)** | -0.027 | 0.033 | -0.092 | 0.037 | 0.406 |
| **Gender** |  |  |  |  |  |
| Female | Ref. |  |  |  |  |
| Male | -0.246 | 0.125 | -0.491 | -0.001 | **0.049** |
| **Nationality** |  |  |  |  |  |
| Italian | Ref. |  |  |  |  |
| Other | -0.505 | 0.644 | -0.762 | 1.771 | 0.434 |
| **Mother tongue** |  |  |  |  |  |
| Italian | Ref. |  |  |  |  |
| Other | -0.043 | 0.650 | -1.320 | 1.234 | 0.947 |
| **Year of study** |  |  |  |  |  |
| 1° | Ref. |  |  |  |  |
| 2° | -0.195 | 0.139 | -0.470 | 0.079 | 0.162 |
| >3° | 0.428 | 0.177 | 0.081 | 0.776 | **0.016** |
| **Perceived economic situation** |  |  |  |  |  |
| Poor or Insufficient | Ref. |  |  |  |  |
| Excellent or Good | -0.158 | 0.229 | -0.607 | 0.292 | 0.491 |
| **Perceived health status** |  |  |  |  |  |
| Poor or Insufficient | Ref. |  |  |  |  |
| Excellent or Good | 0.315 | 0.271 | -0.220 | 0.850 | 0.246 |
| **Healthcare services usage** |  |  |  |  |  |
| Non-users (less than 1 times per year) | Ref. |  |  |  |  |
| Users (at least 1-2 times per year) | 0.177 | 0.131 | -0.080 | 0.435 | 0.177 |
| **Health literacy score** |  |  |  |  |  |
| Inadequate | Ref. |  |  |  |  |
| Problematic | 0.262 | 0.245 | -0.220 | 0.744 | 0.285 |
| Sufficient | 0.292 | 0.301 | -0.299 | 0.883 | 0.331 |

## **Table SXII** Multiple Linear Regression Model of nursing students’ subgroup. Dependent variable: comprehension score (Study 2)

|  | **Adjusted Estimate** | **Standard Error** | **Lower 95% CI** | **Upper 95% CI** | **p-value** |
| --- | --- | --- | --- | --- | --- |
| **Group** |  |  |  |  |  |
| Control group | Ref. |  |  |  |  |
| Intervention group | 0.784 | 0.127 | 0.534 | 1.035 | **<0.001** |
| **Age (years)** | 0.007 | 0.015 | -0.022 | 0.036 | 0.637 |
| **Gender** |  |  |  |  |  |
| Female | Ref. |  |  |  |  |
| Male | -0.197 | 0.178 | -0.548 | 0.153 | 0.269 |
| **Nationality** |  |  |  |  |  |
| Italian | Ref. |  |  |  |  |
| Other | -0.668 | 0.418 | -1.490 | 0.154 | 0.111 |
| **Mother tongue** |  |  |  |  |  |
| Italian | Ref. |  |  |  |  |
| Other | 0.086 | 0.397 | -0.695 | 0.866 | 0.830 |
| **Year of study** |  |  |  |  |  |
| 1° | Ref. |  |  |  |  |
| 2° | 0.505 | 0.145 | 0.221 | 0.789 | **0.001** |
| >3° | 1.013 | 0.177 | 0.665 | 1.361 | **<0.001** |
| **Perceived economic situation** |  |  |  |  |  |
| Poor or Insufficient | Ref. |  |  |  |  |
| Excellent or Good | -0.170 | 0.181 | -0.527 | 0.186 | 0.348 |
| **Perceived health status** |  |  |  |  |  |
| Poor or Insufficient | Ref. |  |  |  |  |
| Excellent or Good | 0.017 | 0.231 | -0.436 | 0.471 | 0.941 |
| **Healthcare services usage** |  |  |  |  |  |
| Non-users (less than 1 times per year) | Ref. |  |  |  |  |
| Users (at least 1-2 times per year) | 0.249 | 0.133 | -0.014 | 0.511 | 0.063 |
| **Health literacy score** |  |  |  |  |  |
| Inadequate | Ref. |  |  |  |  |
| Problematic | -0.407 | 0.239 | -0.878 | 0.063 | 0.090 |
| Sufficient | -0.354 | 0.301 | -0.946 | 0.239 | 0.241 |
